# Detoxification of 5-hydroxymethylfurfural by the *Pleurotus ostreatus* lignolytic enzymes aryl alcohol oxidase and dehydrogenase

**DOI:** 10.1186/s13068-015-0244-9

**Published:** 2015-04-11

**Authors:** Daria Feldman, David J Kowbel, N Louise Glass, Oded Yarden, Yitzhak Hadar

**Affiliations:** Department of Plant Pathology and Microbiology, The R.H. Smith Faculty of Agriculture, Food and Environment, The Hebrew University of Jerusalem, POB 12, Rehovot, 76100 Israel; Department of Plant and Microbial Biology, University of California at Berkeley, 111 Koshland Hall, Berkeley, California 94720 USA

**Keywords:** *Pleurotus ostreatus*, 5-hydroxymethylfurfural (HMF), Aryl-alcohol oxidase, Aryl-alcohol dehydrogenase

## Abstract

**Background:**

Current large-scale pretreatment processes for lignocellulosic biomass are generally accompanied by the formation of toxic degradation products, such as 5-hydroxymethylfurfural (HMF), which inhibit cellulolytic enzymes and fermentation by ethanol-producing yeast. Overcoming these toxic effects is a key technical barrier in the biochemical conversion of plant biomass to biofuels. *Pleurotus ostreatus*, a white-rot fungus, can efficiently degrade lignocellulose. In this study, we analyzed the ability of *P. ostreatus* to tolerate and metabolize HMF and investigated relevant molecular pathways associated with these processes.

**Results:**

*P. ostreatus* was capable to metabolize and detoxify HMF 30 mM within 48 h, converting it into 2,5-bis-hydroxymethylfuran (HMF alcohol) and 2,5-furandicarboxylic acid (FDCA), which subsequently allowed the normal yeast growth in amended media. We show that two enzymes groups, which belong to the ligninolytic system, aryl-alcohol oxidases and a dehydrogenase, are involved in this process. HMF induced the transcription and production of these enzymes and was accompanied by an increase in activity levels. We also demonstrate that following the induction of these enzymes, HMF could be metabolized *in vitro*.

**Conclusions:**

Aryl-alcohol oxidase and dehydrogenase gene family members are part of the transcriptional and subsequent translational response to HMF exposure in *P. ostreatus* and are involved in HMF transformation. Based on our data, we propose that these enzymatic capacities of *P. ostreatus* either be integrated in biomass pretreatment or the genes encoding these enzymes may function to detoxify HMF via heterologous expression in fermentation organisms, such as *Saccharomyces cerevisiae*.

**Electronic supplementary material:**

The online version of this article (doi:10.1186/s13068-015-0244-9) contains supplementary material, which is available to authorized users.

## Background

Ethanol biofuel derived from lignocellulosic biomass is a viable alternative to fossil fuel-based transportation fuels [1]. Unlike first-generation biofuels, which are produced from corn, lignocellulosic biofuels do not compete with food-derived ethanol and can be made from abundant and renewable plant biomass sources [2]. Complex carbohydrates in the plant cell wall, such as cellulose and hemicellulose, are closely associated with lignin, and pretreatment using thermo and/or chemical processes is necessary to increase their availability for enzymatic hydrolysis and fermentation [1,2]. In spite of the necessity for pretreatment of plant biomass, it can be a rate-limiting step in fermentation due to the production of inhibitory compounds, particularly furans, such as 5-hydroxymethylfurfural (HMF) and furfural and other phenolic compounds [3-5]. The level of furans produced during pre-treatment varies according to the type of raw material and the pretreatment procedure [6-9].

Furfural and HMF are the most potent inhibitors generated via pretreatment [10-13]. The effect on the growth rate on *Saccharomyces cerevisiae* and the subsequent decrease in the fermentation rate is higher for furfural than for HMF, but the effect of HMF lasts longer [14]. Several mechanisms may explain the inhibition effects on yeast growth and ethanol fermentation by exposure to furans. *In vitro* experiments and crude cell extract measurements showed that HMF directly inhibited alcohol dehydrogenase, pyruvate dehydrogenase, and aldehyde dehydrogenase. This inhibition of enzyme activity occurs along with the re-direction of yeast energy to repair the damage caused by furans and by reduced intracellular ATP and NAD(P)H levels, either by enzymatic inhibition or consumption/regeneration of co-factors [15].

Microarray-based expression studies in *S. cerevisiae* identified more than 300 genes that were expressed at significantly higher levels after exposure to furans. Based on these results, it was concluded that furan degradation is catalyzed by multiple aldehyde reductases and tolerance to these compounds can be conferred by enhanced expression of members of pleiotropic drug resistance genes [16,17]. An HMF metabolic conversion product was isolated and identified as 2,5-bis-hydroxymethylfuran (HMF alcohol) [18,19], which is catalyzed by various aldehyde reductases in the presence of NAD(P)H as a co-factor [16]. The bacterium *Cupriavidus basilensis* was shown to grow on HMF as a sole carbon source and harbors a gene cluster involved in HMF metabolism. In *C. basilensis*, HMF oxidation activity [20], was catalyzed by HMF oxidase (HMFO) encoded by *HmfH* [20,21]. The corresponding homologue was cloned from a *Methylovorus* sp. strain MP688, and an HMFO enzyme was shown to oxidize HMF to 5-(hydroxymethyl)furoic acid (HMF acid) and to 2,5-furandicarboxylic acid (FDCA), during which H_2_O_2_ was generated [21]. The fungus *Amorphotheca resinae* ZN1 was isolated from pretreated corn stover and was shown to also degrade HMF, both to HMF alcohol and HMF acid, under aerobic conditions [22].

Overcoming the effects of pretreatment toxicity in biofuel-producing organisms, such as yeast, is a key technical challenge in the biochemical conversion of biomass feed-stocks to biofuels. The basidiomycete *Pleurotus ostreatus*, a white-rot fungus, can efficiently degrade lignin, cellulose, and hemicellulose [23-25], thus providing potential tools for biological pretreatment in biofuel production [26]. Furthermore, *P. ostreatus* has been shown to degrade a wide variety of phenolic compounds including those that are inhibitory to *S. cerevisiae* [27]. Hence, we hypothesized that *P. ostreatus* may metabolize HMF by enzymatic pathways that are specific and/or abundant in white rot fungi.

In this study, we demonstrate, for the first time, that *P. ostreatus* can bio-convert HMF to HMF alcohol and FDCA, thus detoxifying the compound. We show that exposure to HMF increases the expression, translation, and activity of enzymes involved in the ligninolytic system, including aryl-alcohol oxidases and a dehydrogenase. Both enzyme families can specifically bio-convert HMF and contribute to the tolerance of *P. ostreatus* to HMF.

## Results

### HMF is bio-converted by *P. ostreatus*

**Figure 1:**
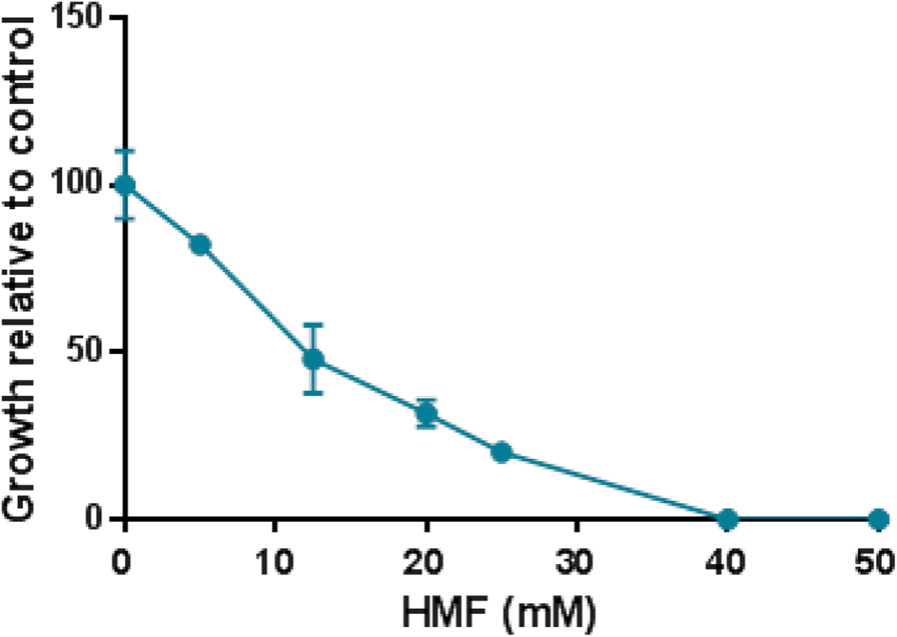
**Relative growth of** ***P. ostreatus*** **in the presence of different concentrations of HMF.** Different concentrations of HMF were added to GP solid media and linear growth of *P. ostreatus* was measured relative to a control lacking HMF. Bars indicate standard errors.

In order to explore the effect of HMF on *P. ostreatus*, we first examined the tolerance of the fungus toward the compound. The growth of *P. ostreatus* PC9 on a solid glucose-peptone (GP) medium supplemented with different concentrations of HMF was measured. Under these conditions, the IC_50_ of HMF to *P. ostreatus* was 12.5 mM (Figure 1), which is significantly higher than the value reported for *S. cerevisiae* (viability percent was log_10_ = 10 on YPD) [28]. The fact that *P. ostreatus* is more tolerant than *S. cerevisiae* to the compound suggests that it may harbor more efficient mechanisms to metabolize HMF or otherwise avoid the toxic effects of this compounds.

To determine whether *P. ostreatus* can metabolize HMF, we conducted experiments in liquid GP medium, in which the fungus was cultured for 5 days to accumulate biomass prior to the addition of HMF 30 mM. Control treatments were identical, excluding the HMF amendment. The amount of HMF and metabolites were monitored colorimetrically and verified by gas chromatography–mass spectrometry (GC-MS) analyses using standards. After 8 h, the extracellular concentration of HMF was reduced by approximately 10%, 24 h marked the point of 50% reduction, and complete transformation occurred after 48 h. HMF alcohol was detected after 8 h and remained in the media for 48 h (Additional file 1). From the oxidation derivatives of HMF, we only detected FDCA after 24 h, but not after 48 h (Additional file 1).

To determine if the bio-transformation of HMF by *P. ostreatus* also results in reducing its toxic effects on yeast, we preformed experiments in which HMF-amended medium was subjected to *P. ostreatus* detoxification prior to cultivation of *S. cerevisiae* on the spent medium. Yeast grown in the presence of 30 mM HMF for 30 h accumulated only 30% of the biomass as compared to control cultures lacking HMF. By contrast, when *S. cerevisiae* was inoculated into spent medium from *P. ostreatus* cultures grown for 8 or 24 h in the presence of HMF, growth of the culture was elevated to 50% and to 85%, respectively. No inhibition of yeast growth was observed in spent HMF-amended medium after 48 h of *P. ostreatus* growth (Additional file 2). These results are in agreement with the level of transformation of HMF by *P. ostreatus* at those time points and suggest that the fungus has the potential to be used for reducing the toxic effects of HMF and may serve as a safe pretreatment amendment.

### Abundance of aryl-alcohol oxidases and dehydrogenases increases in the presence of HMF

**Figure 2:**
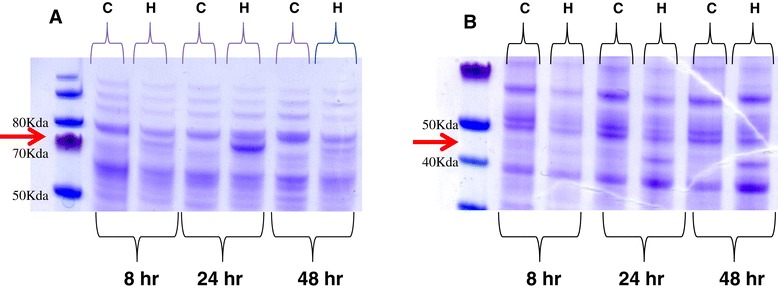
**Profiles of secreted and cellular** ***P. ostreatus*** **proteins obtained from cultures grown in the presence of HMF.** Secreted **(A)** and cellular **(B)** proteins were extracted from *P. ostreatus* 8, 24, and 48 h after addition of 30 mM of HMF to the media. The proteins were resolved by SDS-PAGE 4% to 12%. C: control cultures without HMF, H: cultures exposed to HMF. Arrows point to major visible difference in the profiles.

To investigate the enzymatic processes involved in HMF transformation by *P. ostreatus*, a time course experiment in which changes in the profiles of secreted and intracellular proteins as a result of HMF exposure was monitored. Proteins were concentrated from the extracellular fraction at different stages of HMF transformation (8, 24, and 48 h after HMF addition to the media, corresponding to approximately 5%, 50%, and 100% transformation, respectively). Surprisingly, a significant increase in the abundance of an approximately 75-kDa secreted protein was evident from samples at the beginning of the transformation process (only 8 h after HMF addition). Its abundance peaked at a time corresponding to 50% HMF transformation and diminished once all the HMF was detoxified (Figure 2). The approximately 75-kDa band was sequenced and identified as having a high coverage and score of 6 aryl-alcohol oxidase proteins (AAO; EC 1.1.3.7): 69649, 82653, 93955, 114510, 116309, and 121882, designated *aao1-6,* respectively. AAOs are secreted peroxide-producing flavoenzymes and members of the glucose-methanol-choline oxidase (GMC) oxidoreductase superfamily [29-31]. *P. ostreatus* has a large AAO, a gene family consisting of at least 36 genes [32]. Phylogenetic analysis of the AAOs in *P. ostreatous* revealed that they cluster into two groups (Additional file 3).

Among the intracellular proteins obtained from the same cultures, a significant increase in an approximately 45-kDa protein band was observed, whose abundance also correlated with the extent of HMF detoxification. An increase in the abundance of the approximately 45-kDa protein was observed at the early stage of transformation, peaking during the mid-phase; in contrast to the AAOs, the levels of this protein remained constant even when HMF transformation was completed (Figure 2). The sequence of the approximately 45-kDa band identified it as an aryl alcohol dehydrogenase (AAD; EC 1.1.1.90): 75413, which we designated *aad1*. AADs have been shown to reduce alcohols and acids to aldehydes and alcohols, respectively, using nicotinamide adenine dinucleotide phosphate (NADPH) as a co-factor [33]. As HMF is both an aryl alcohol and an aldehyde (Additional file 1), the increase in the abundance of these extracellular and intracellular proteins suggests that there is an involvement of both AAOs and AADs in the response of *P. ostreatus* to HMF.

### HMF induces elevated expression levels of AAO and AAD

**Figure 3:**
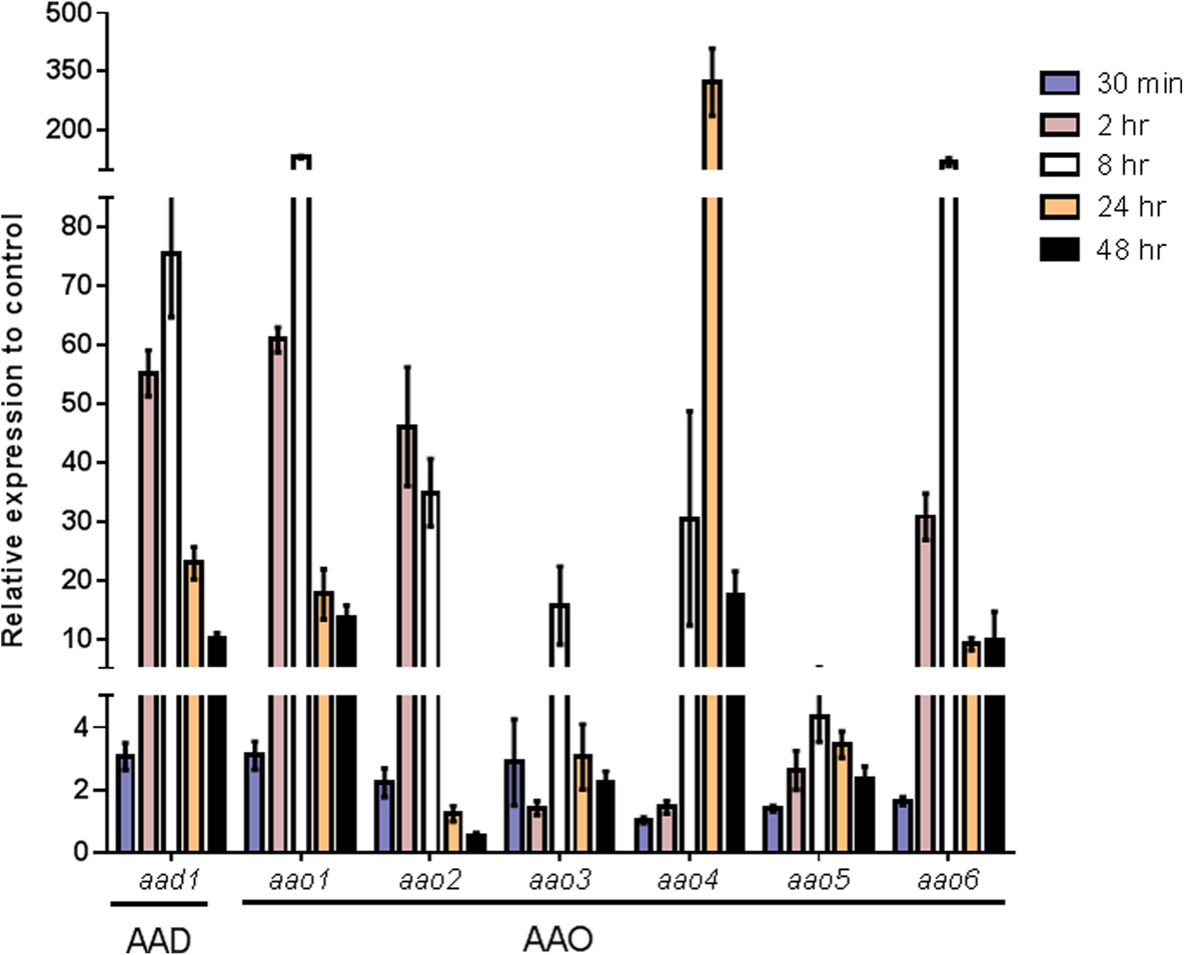
**Time course expression of** ***P. ostreatus aao1-6*** **and** ***aad1*** **genes following the addition of HMF.** The expression levels of *aao1-6*, *aad1*, and *vp1* were monitored by real-time RT-PCR (for primers information see Additional file 4). RNA was extracted from *P. ostreatus* at different time points (0.5, 2, 8, 14, and 48 h) after addition of 30 mM HMF to the media. The expression levels calculated relative to *β-tubulin*, as the endogenous control, and represent the expression relative to control without HMF addition. Bars indicate standard errors.

In order to verify whether the observed AAO and AAD protein accumulation is a consequence of alteration in the expression of the corresponding genes, we constructed specific primers to monitor their expression levels by quantitative real-time polymerase chain reaction (RT-PCR). RNA was extracted from *P. ostreatus* hyphae at different time points following exposure to HMF. Following the kinetics of expression, we observed a significant and substantial increase in all AAO and AAD mRNA levels (Figure 3), with three patterns of expression. The first group (*aao1-3* and *aad1*) was rapidly transcribed following exposure to HMF and expression levels increased at least twofold relative to the control as early as 30 min after exposure to the furan. The second group (*aao5-6*) showed increased expression levels after 2 h of exposure to HMF. Both these groups, the maximum expression change ranged from 4- to 150-fold, which was observed following 8 or 24 h of exposure to HMF. After this time period, transcript abundance of both of these groups of genes declined. *aao4* exhibited a third expression pattern characterized by a slower increase in expression, starting only 8 h after exposure to HMF. Although the observed increase in the rate of *aao4* expression was slower than the other groups of genes, it exhibited the most dramatic change (approximately 300-fold) in its expression level, at 24 h after HMF exposure.

The differential expression pattern of the *aao*s and *aad* genes suggest that they may have different roles in the molecular response of *P. ostreatus* to HMF, as the expression kinetics corroborate with the observed AAO and AAD protein levels (Figure 2).

### HMF is a substrate of both AAOs and AADs

**Figure 4:**
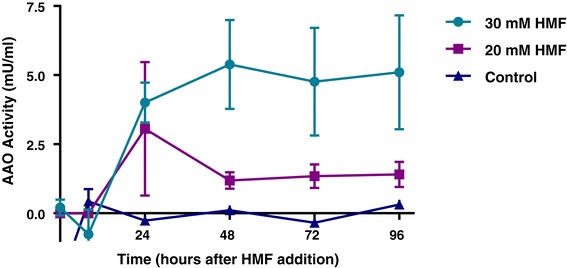
***In vitro*** **AAO activity is increased in the extracellular fraction of** ***P. ostreatus*** **after addition of HMF to the medium.** The activity of AAO following 1 mM veratryl alcohol addition as a substrate was monitored in free cell extracts of *P. ostreatus*, at different time points after addition of 30 or 20 mM HMF to the media. Bars indicate standard errors.

The enzymatic systems of AAO and AAD families are not substrate-specific, and these proteins are responsible for the degradation and detoxification of a variety of organic compounds [33,34]. Since HMF has both aldehyde and alcohol functional groups (Additional file 1), we assumed that, in addition of being inducer, HMF could be a potential substrate of these enzymes. To explore whether these enzyme families could directly reduce or oxidize HMF to its derivatives, we further monitored their enzymatic activities *in vitro*. AAOs oxidize alcohols and aldehydes to acids and alcohols, respectively [29,33]. Enzyme activity was measured using a specific well-studied substrate, veratryl alcohol (3,4-dimethoxybenzyl alcohol) [35] in samples prepared from *P. ostreatus* culture media at different time points of HMF exposure. A significant increase in the activity of AAO was observed 24 h after the addition of 30 mM HMF and remained at a level of approximately 5 mU/ml even 4 days after HMF was added (Figure 4). The enzyme activity level was found to be dependent on HMF concentration in the culture. In fact the enzyme activity in the presence of HMF 20 Mm was only 2 mU/ml. In the *P. ostreatus* control cultures that were not exposed to HMF, activity was detected 7 days later (12 days after inoculation) and reached a higher level (1,250 mU/ml), which represents the anticipated increase in enzyme activity at that late growth phase [30,33]. Thus, an increase in AAO protein abundance was correlated with an increase in corresponding enzymatic activities (as determined colorimetrically).

**Figure 5:**
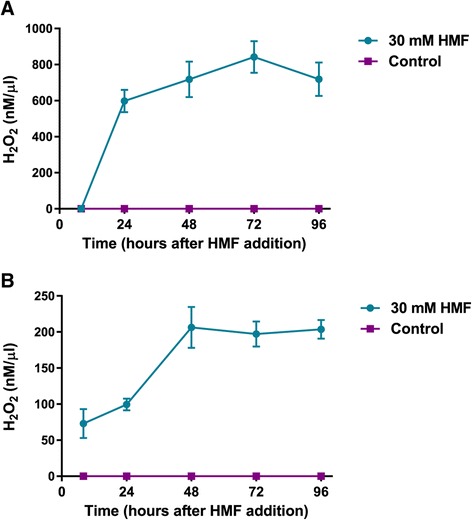
***In vitro*** **generation of peroxide is increased in the extracellular fraction of** ***P. ostreatus*** **after addition of HMF to the medium.** Concentration of H_2_O_2_ generated during activity *in vitro* with 1 mM veratryl alcohol **(A)** or 10 mM HMF **(B)** over time in free cell extracts of *P. ostreatus*. The measurements were performed at different time points after addition of 30 mM HMF to the medium. Bars indicate standard errors.

Secreted AAOs oxidize alcohols and aldehydes while generating H_2_O_2_. We therefore also monitored peroxide production as another indication for AAO activity [21,36]. Indeed, an increase in H_2_O_2_ concentration was observed in the samples containing veratryl alcohol as a substrate, 24 h after HMF addition to the fungal culture. Furthermore, peroxide levels in the reaction mixture were maintained at approximately 700 nM/μl, even 4 days after exposure. By contrast, at the same time points, H_2_O_2_ was not detected in control cultures (Figure 5A). These results are in agreement with the changes in AAO activity levels described above (Figure 4). When HMF was introduced as a substrate to the assay mixture, peroxide levels increased to approximately 70 nM/μl as soon as 8 h after exposure (Figure 5B). The maximum level of H_2_O_2_ detected (approximately 200 nM/μl) occurred 2 to 4 days after HMF addition to the fungal culture. These results could be explained by changes in preferential expression of the different AAOs (Figure 3), suggesting that AAOs whose expression is induced shortly after exposure to HMF may have a higher affinity for HMF than for veratryl alcohol.

**Figure 6:**
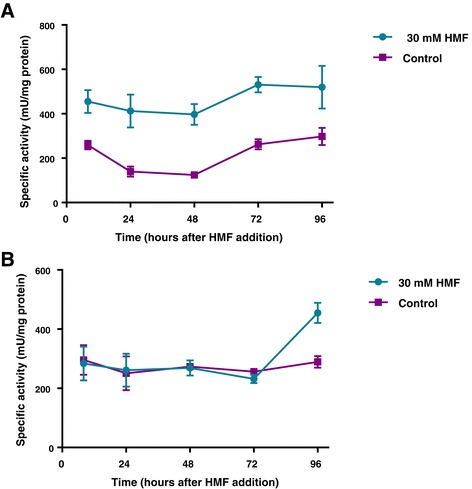
**Specific activity coupled with NAD(P)H in free cell extracts of** ***P. ostreatus*** **after addition of HMF to the medium.** Depletion of NADPH **(A)** or NADH **(B)** was monitored over time *in vitro* with cell extracts of *P. ostreatus* with 10 mM HMF as a substrate. The measurements were performed at different time points after addition of 30 mM HMF to the culture.

HMF can be reduced in the intracellular fraction in *S. cerevisiae* by either NADPH or nicotinamide-adenine dinucleotide (NADH) as co-factors in the reaction [16,19,37]. In *P. ostreatus*, we observed an increase in intracellular AAD expression and protein accumulation (Figures 2 and 3), as well as an accumulation of the AAD reaction product, HMF-alcohol (Additional file 1). We therefore examined whether *P. ostreatus* cell free extracts can directly reduce HMF, by monitoring NAD(P)H depletion in cell extracts of *P. ostreatus* exposed to HMF. Specific activity coupled with NADPH doubled in samples extracted from *P. ostreatus* 8 h after addition of HMF and was maintained over time at a steady level of 400 to 500 mU/mg protein (Figure 6A). Specific activity coupled with NADH did not alter as a response to exposure to HMF and remained at the level of approximately 250 mU/mg protein. However, after 4 days, activity increased by 80% (Figure 6B), which may suggest an additional secondary response to the compound. We conclude that AAD can directly reduce HMF while using NADPH as a preferred co-factor.

## Discussion

*P. ostreatus* is a commercially important edible, ligninolytic white-rot filamentous basidiomycete species. It can be easily cultivated on a variety of organic substrates, including agricultural wastes. Hence, it has potential to be harnessed as a pretreatment process for recycling of lignocellulosic substrates, in view of biofuel production [24-26,38]. In this study, we probed the ability of *P. ostreatus* to tolerate and detoxify HMF, a compound that imposes a rate-limiting step in thermo-chemical pretreatment [5] via inhibition of efficient biomass utilization [3,4].

This is the first time that the ability of a white-rot fungus to tolerate HMF was explored. We found that *P. ostreatus* is more tolerant to HMF than *S. cerevisiae*. Furthermore, *P. ostreatus* has the capability in metabolizing HMF to HMF alcohol and FDCA. While HMF alcohol remained in the media and was not metabolized, FDCA was detected only at one time point, suggesting that the fungus can process it further. As *S. cerevisiae* has been shown to degrade HMF to HMF alcohol [18,19], but not to HMF acid or FDCA, we hypothesized that other, perhaps unique, oxidative pathways may be involved in *P. ostreatus*’ capability to do so.

The secreted AAOs, which are peroxide-producing flavoenzymes that belong to GMC oxidoreductase superfamily [29], oxidized HMF and generated H_2_O_2_ during the process (Figure 5). We speculate that it may catalyze a reaction similar to HMFO from *C. basilensis* [20,21]. HMFO also belongs to the GMC superfamily and can oxidize HMF to HMF acid and FDCA [21]. The changes in AAO expression patterns (Figure 3), combined with HMF being the preferred substrate over veratryl alcohol (Figure 5), along with the large number of AAOs in the *P. ostreatus* genome [32], lead us to suspect that the different AAOs, induced at different times after exposure, have different substrate specificities. Since no AAO homologues have been found in *S. cerevisiae*, heterologous expression of the AAO-encoding genes may be a means to improve yeast tolerance to HMF. Such an approach has already proved feasible by the expression of a laccase gene from the white-rot fungus *Trametes versicolor* in *S. cerevisiae*, to produce a strain that exhibited increased resistance to phenolic inhibitors present in lignocellulose hydrolysates [5,39]. Flavin-based redox enzymes, such as AAOs, have gained enormous interest and importance in the development of biosensors and production of industrially useful carbonyl compounds [40], suggesting another biotechnological potential for the described AAOs from *P. ostreatus*.

Within the cell, transformation of HMF was catalyzed by AAD, an intracellular reducing enzyme that uses NADPH as a co-factor [33] (Figure 6). The pathway involved is probably similar to the conversion of HMF to HMF alcohol which was shown to be catalyzed by various aldehyde reductases in *S. cerevisiae* [16]. The link between AAD and HMF was described in yeast, where expression of two AADs (AAD4, AAD14) was increased in HMF-grown cells [41]. We suggest that the *P. ostreatus aad1* can be another potential candidate whose heterologous expression may enhance HMF degradation in yeast, while improving tolerance to this furan during pretreatment. The preference of yeast enzymes toward co-factors varies between NADPH to NADH [16]. As predicted, the reaction catalyzed by AAD was increased with NADPH (Figure 6A). Since basal levels of HMF transformation was coupled with NADH as well (Figure 6B), we suspect that hydrolases and reductases other than AAD1 are also involved in the process. Surprisingly, a significant increase in the transformation with NADH occurred 4 days after HMF was added to the media (Figure 6B), which supports the involvement of an additional, yet unknown, transformation reaction or pathway for HMF in *P. ostreatus*.

## Conclusion

**Figure 7:**
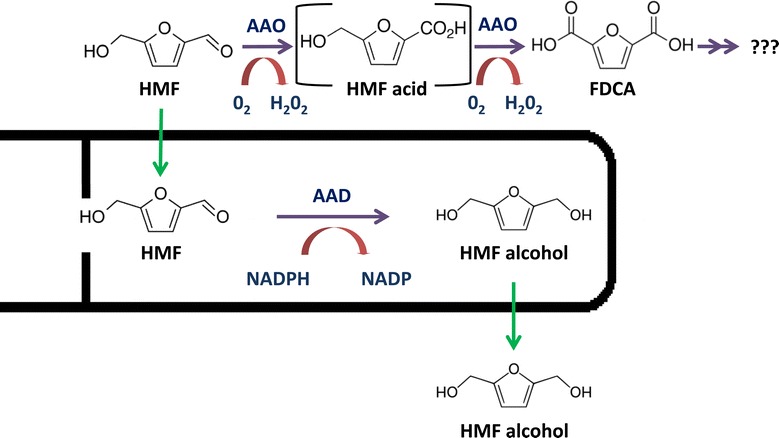
**Scheme for the enzymatic degradation of HMF by** ***P. ostreatus.*** HMF is metabolized extracellularly by AAO to FDCA, which generates H_2_O_2_. These reactions probably involve an intermediate conversion of HMF to HMF acid and further conversion to unknown products. When HMF enters the cell, it is reduced by AAD with NADPH as a co-factor and metabolized to HMF alcohol. HMF alcohol can be secreted and accumulates extracellularly.

Most of the research on *P. ostreatus* has focused on its ability to degrade lignin [25,38], as a means for providing an alternative biological pretreatment of biomass in biofuel production [26]. Here, we have described and subsequently analyzed the ability of *P. ostreatus* to bio-convert a pre-treatment toxic byproduct, HMF, by enzymes usually associated with the lignin degradation complex (Figure 7). Based on our findings, we suggest that *P. ostreatus* can be potentially integrated as part of the physical and chemical pretreatment process, either by direct use of the fungus or its enzymes or by mining the genetic pool of this white-rot fungus for genes to be heterologously expressed in yeast or other biofuel-producing microorganisms. Such a strategy has already been employed to facilitate direct fermentation of cellodextrins by yeast, where *Neurospora crassa* was used as a gene pool and its two cellodextrin transporters were introduced into *S. cerevisiae* along with an intracellular β-glucosidase, which subsequently improved cellobiose fermentation [42,43]. Expanding and/or combining the resources available from various fungal gene pools may well prove beneficial in engineering yeast strains tailored to challenge rate limiting steps in biofuel production.

## Methods

### Fungal growth and experimental conditions

*P. ostreatus* monokaryon strain PC9 (Spanish Type Culture Collection accession number CECT20311), which is a protoclone derived by de-dikaryotization of the commercial dikaryon strain N001 (Spanish Type Culture Collection accession number CECT20600) [44], was used throughout this study.

Fungal strains were grown and maintained in GP medium (2% glucose, 0.5% peptone (Difco, Franklin Lakes, NJ, USA), 0.2% yeast extract (Difco, Franklin Lakes, NJ, USA), 0.1% K_2_HPO_4_, and 0.05% MgSO_4_ · 7H_2_O). When required, 1.5% agar was added. The gene and protein expression as well as activity assays were conducted in samples of fungal biomass or cell free extracellular extracts taken from liquid cultures that were maintained in stationary 100 ml Erlenmeyer flasks containing 10 ml of media. The fungus was grown for 5 days to accumulate biomass, after which HMF (Sigma-Aldrich, St. Louis, MO, USA) was added to the media to obtain concentrations of 20 to 30 mM. All experiments were accompanied by controls that lacked the chemical amendment. The inoculum for all growth conditions was one disk (5 mm diameter) of mycelium obtained from the edge of a young colony grown on solid medium and positioned at the center of the Petri dish or a flask. Cultures were incubated at 28°C in the dark.

*S. cerevisiae* strain CBS8066 was maintained on 10 g/l yeast extract, 20 g/l peptone, 20 g/l glucose, and 20 g/l agar (YPD agar) or on 10 g/l yeast extract, 20 g/l peptone, and 20 g/l glucose (YPD). For the spent medium experiments, the cultures were grown on spent *Pleurotus* medium (GP) supplemented initially with 30 mM HMF. The cultures were grown in an orbital shaker (180 rpm) at 30°C under aerobic conditions. The growth was monitored at 600 nm using the Synergy 2 Multi-Mode Microplate Reader (BioTek, Winooski, VT, USA). The assay was performed in triplicate, and an average reading was plotted.

### Gene expression analyses

Total RNA was extracted from culture biomass, first ground under liquid nitrogen with mortar and pestle, then homogenized with QIA shredder spin columns (Qiagen, Hilden, Germany) and purified from the lysate using the RNeasy Plus Mini Kit (Qiagen, Hilden, Germany). cDNA was synthesized using the High Capacity cDNA Reverse Transcription Kit (Applied Biosystems, Carlsbad, CA, USA). Gene expression analyses were performed on an ABI StepOne Real-Time PCR Sequence Detection System and software (Applied Biosystems, Foster City, CA, USA), using Power SYBR Green PCR Master Mix (Applied Biosystems, Foster City, CA, USA). The PCR volume was 10 μl, using 20 ng of total cDNA and 300 nM oligonucleotide primers (Additional file 4). The thermal cycling conditions were as follows: an initial step at 95°C for 20 s and 40 cycles at 95°C for 5 s, 60°C for 30 s, followed by a denaturation step to verify the absence of unspecific products or primer dimmers. The *β-tubulin* (ID: 117235) gene was used as the endogenous control. The primer efficiency levels of the genes were with the range of 90% to 110%. Amplification data were compared on the basis of the ΔΔCT method and presented as 2^-ΔΔCT^. Data was normalized with respect to *β-tubulin* and calculated where ΔCT = CT *target gene* − CT *β-tubulin*, and then ΔΔCT = ΔCT *treatment with 30 mM HMF* − ΔCT *control without HMF.*

### Protein expression profiles

For extracellular protein analyses, culture fluids were filtered through Whatman No. 1 filter paper followed by 0.45-μm mixed cellulose ester filter paper (Whatman, Buckinghamshire, UK). The sample was then concentrated using a 10-kDa cutoff PM-10 membrane (Millipore, Amicon Division, Billerica, MA, USA) and treated with cOmplete (Roche Applied Science, Mannheim, Germany), after concentration. For intracellular protein extraction, mycelial samples were frozen in liquid nitrogen, pulverized, and suspended in lysis buffer (1 M sorbitol, 10 mM HEPES (pH 7.5), 5 mM EDTA, 5 mM EGTA, 5 mM NaF, 0.1 M KCl, 0.2% Triton X-100, cOmplete (Roche Applied Science, Mannheim, Germany). The samples were homogenized by ten strokes of pestle A in a Dounce homogenizer. The homogenates were centrifuged for 40 min at 10,000 × *g* at 4°C, and the supernatants were recovered and stored at −70°C until analyzed.

The protein concentration was determined using the BioRad protein assay kit (BioRad, Hercules, CA, USA). The proteins were separated on a NuPAGE 4% to 12% Bis-Tris gel in MES-SDS running buffer (Invitrogen, Grand Island, NY, USA) and visualized with Coomassie R-250 (0.125%). The sample was subsequently analyzed by HPLC/mass spectrometry/mass spectrometry (LC-MS/MS) in an Orbitrap (Thermo Scientific, Waltham, MA, USA) mass spectrometer and identified by Sequest 3.31 software against the JGI genome database of *P. ostreatus* PC9 v1.0 (http://genome.jgi-psf.org/PleosPC9_1/PleosPC9_1.home.html) at The Smoler Proteomics Center of The Israel Institute of Technology (Technion).

### Enzymatic activity assays

AAO activity (AAO): the activity was assayed spectrophotometrically, as the oxidation of veratryl alcohol (3,4-dimethoxybenzyl) to veratraldehyde, monitored at 310 nm (*ε*_310_ = 9,300 M^−1^ cm^−1^). The reaction mixtures contained 1 mM veratryl alcohol in 50 mM potassium phosphate, pH = 6. The assay was conducted in a volume of 200 μl in microtiter plates at 30°C, using the Synergy 2 Multi-Mode Microplate Reader (BioTek, Winooski, VT, USA). An enzyme unit was defined as the amount enzyme producing 1 μmol of product per minute.

AAO activity coupled with H_2_O_2_: This assay was based on a highly sensitive and stable probe for H_2_O_2_, 10-acetyl-3,7-dihydroxyphenoxazine (Amplex Red reagent). The Amplex Red™ kit assay (Invitrogen, Carlsbad, CA, USA) was performed on each sample, according to the manufacturer’s instructions. The filtrate was added to 50 mM potassium phosphate, pH = 6 with 1 mM of veratryl alcohol or 10 mM HMF, to a total volume of 50 μl. The samples were placed in microtiter plates, and Amplex Red reaction mixture (50 μl) was added to each well. The reaction was incubated for 3.5 h at 30°C, after which fluorescence (conversion of the reagent toresorufin) was measured using the Synergy 2 Multi-Mode Microplate Reader (BioTek, Winooski, VT, USA). Excitation and emission were at 540 ± 20 and 590 ± 25 nm, respectively.

AAD activity: The mycelial samples were disrupted using a Bead Beater (BioSpec Products, Inc, Bartlesville, OK, USA) in 500 μl of 100 mM potassium phosphate, pH = 7. The homogenates were centrifuged for 1 min at 4,000 × *g* at 4°C. The protein concentration of the clear lysate was determined using the BioRad protein assay kit (BioRad, Hercules, CA, USA), and 30 μl was used per each reaction. The activity was assayed spectrophotometrically, as NAD(P)H was added to a concentration of 30 μM and monitored at 340 nm (*ε*_340_ = 6,220 M^−1^ cm^−1^) and 10 mM HMF with 100 mM potassium phosphate, pH = 7. The assay was conducted in a total volume of 600 μl at 30°C, and changes in absorption were monitored for 15 min using a spectrophotometer (Biomate 3, Thermo Scientific, Waltham, MA, USA). An enzyme unit was defined as the amount enzyme producing 1 μmol of product per minute and divided by mg of protein.

### Protein accession numbers and phylogeny

For phylogenetic analysis, protein sequences were obtained from the JGI genome database of *P. ostreatus* PC9 v1.0 (http://genome.jgi-psf.org/PleosPC9_1/PleosPC9_1.home.html) using blastp algorithm. The phylogenetic tree was generated using phylogeny.fr [45].

### Chemical analysis

The samples were analyzed using a GC-MS apparatus which consisted of a gas chromatograph (Agilent 7890A, Agilent Technologies, Santa Clara, CA, USA) coupled to the mass selective (Agilent 5975C MSD) detector. The compounds were separated on a BPX-5 capillary column (30 m × 0.25 mm, 0.25 μm, SGE). Helium was used as a carrier gas at a 1.3 ml/min flow rate. Prior to analysis, the samples (150 μl) were evaporated upon dry nitrogen at 50°C and derivatized with 100 μl trimethylsilylation reagent which consisted of pyridine, BSA, and TMCS (20:20:1). Analytical equipment was controlled, and data was analyzed using MassHunter Acquisition and Data Analysis software (Agilent). Analytical standards of HMF and FDCA were purchased from Sigma-Aldrich (St. Louis, MO, USA). HMF acid and HMF alcohol were purchased from Toronto Research Chemicals Inc (North York, Canada).

## Additional files

Additional file 1:
**Summary of the GC-MS analysis of** ***P. ostreatus*** **spent medium after growth in the presence of 30 mM HMF.** The molecules were identified using standards. ND (not detected), +++ (high concentration), ++ (medium concentration), and + (low concentration). Additional file 2:
**Growth of** ***S. cerevisiae*** **cultures on spent medium of** ***P. ostreatus*** **grown in the presence of HMF.** Yeast cultures were inoculated into spent *P. ostreatus* media, initially supplemented with 30 mM of HMF. Yeast growth was monitored for 30 h at 600 nm. Control **(A)** or after an addition of 30 mM of HMF **(B)**. Additional file 3:
**Phylogenetic analysis of the AAO family in** ***P. ostreatus.*** Phylogenetic analysis based on the protein sequences of AAOs from *P. ostreatus.* Green represents proteins whose abundance increased after HMF addition to the media. Blue represents additional genes whose expression was induced (as determined by real-time PCR; data not shown) 24 h after HMF was added to the medium. Additional file 4:
**Primer information.**

## Abbreviations

AAD: aryl-alcohol dehydrogenase
AAO: aryl-alcohol oxidase
FDCA: 2,5-furandicarboxylic acid
GP: glucose-peptone
HMF: 5-hydroxymethylfurfural
HMF acid: 5-(hydroxymethyl)furoic acid
HMF alcohol: 2,5-bis-hydroxymethylfuran
NADH: nicotinamide-adenine dinucleotide
NADPH: nicotinamide adenine dinucleotide phosphate
