# Supplementary figures and images for: Detoxification of 5-hydroxymethylfurfural by the Pleurotus ostreatus lignolytic enzymes aryl alcohol oxidase and dehydrogenase
Source: Biotechnol Biofuels. 2015 Apr 11;8:63. doi: 10.1186/s13068-015-0244-9 (PMC4403834; doi:10.1186/s13068-015-0244-9)

## Slide 1
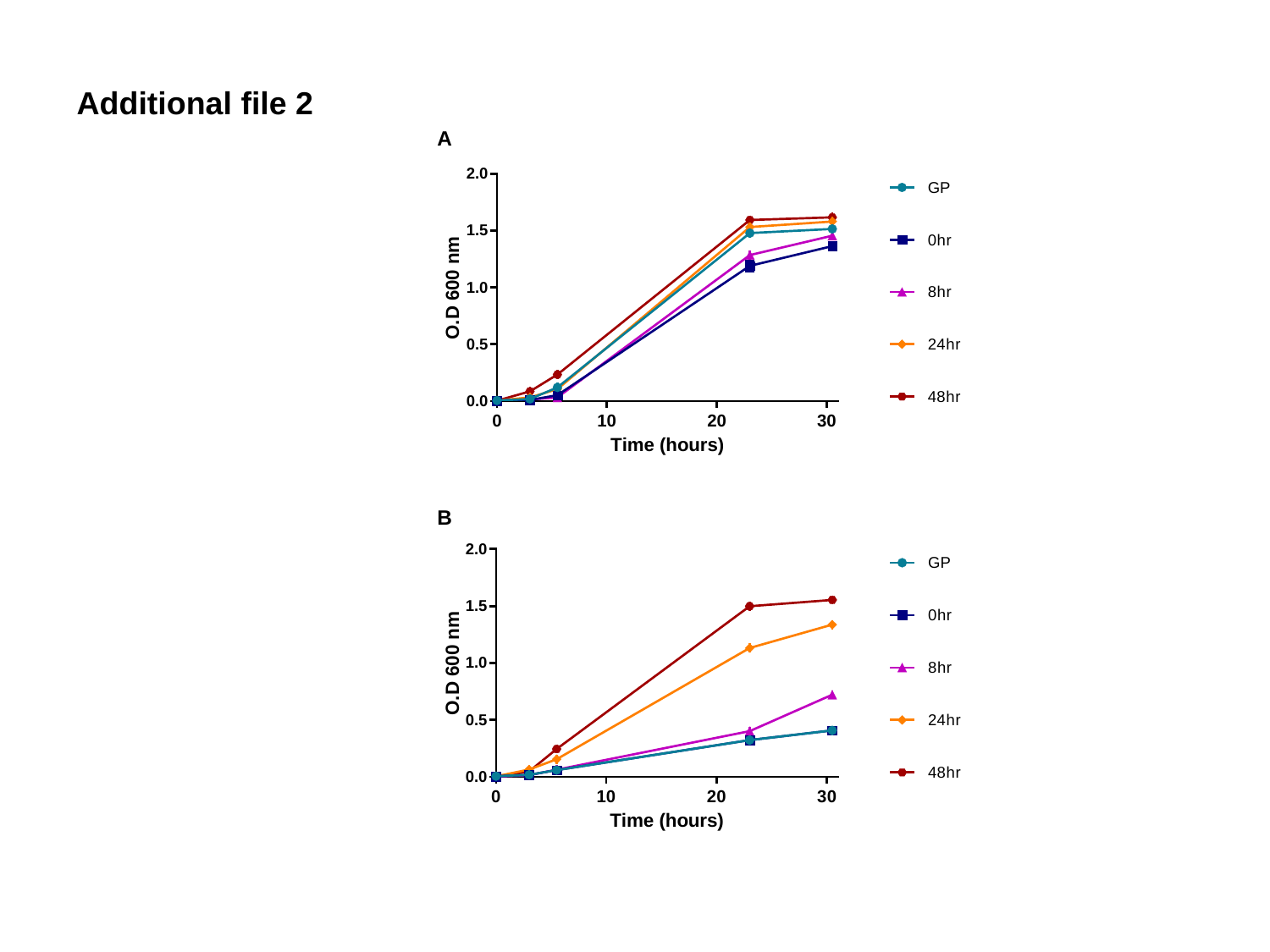

Additional file 2

Supplement: Additional file 2: — Growth of S. cerevisiae cultures on spent medium of P. ostreatus grown in the presence of HMF. Yeast cultures were inoculated into spent P. ostreatus media, initially supplemented with 30 mM of HMF. Yeast growth was monitored for 30 h at 600 nm. Control (A) or after an addition of 30 mM of HMF (B). [file 13068_2015_244_MOESM2_ESM.pptx]

## Slide 1
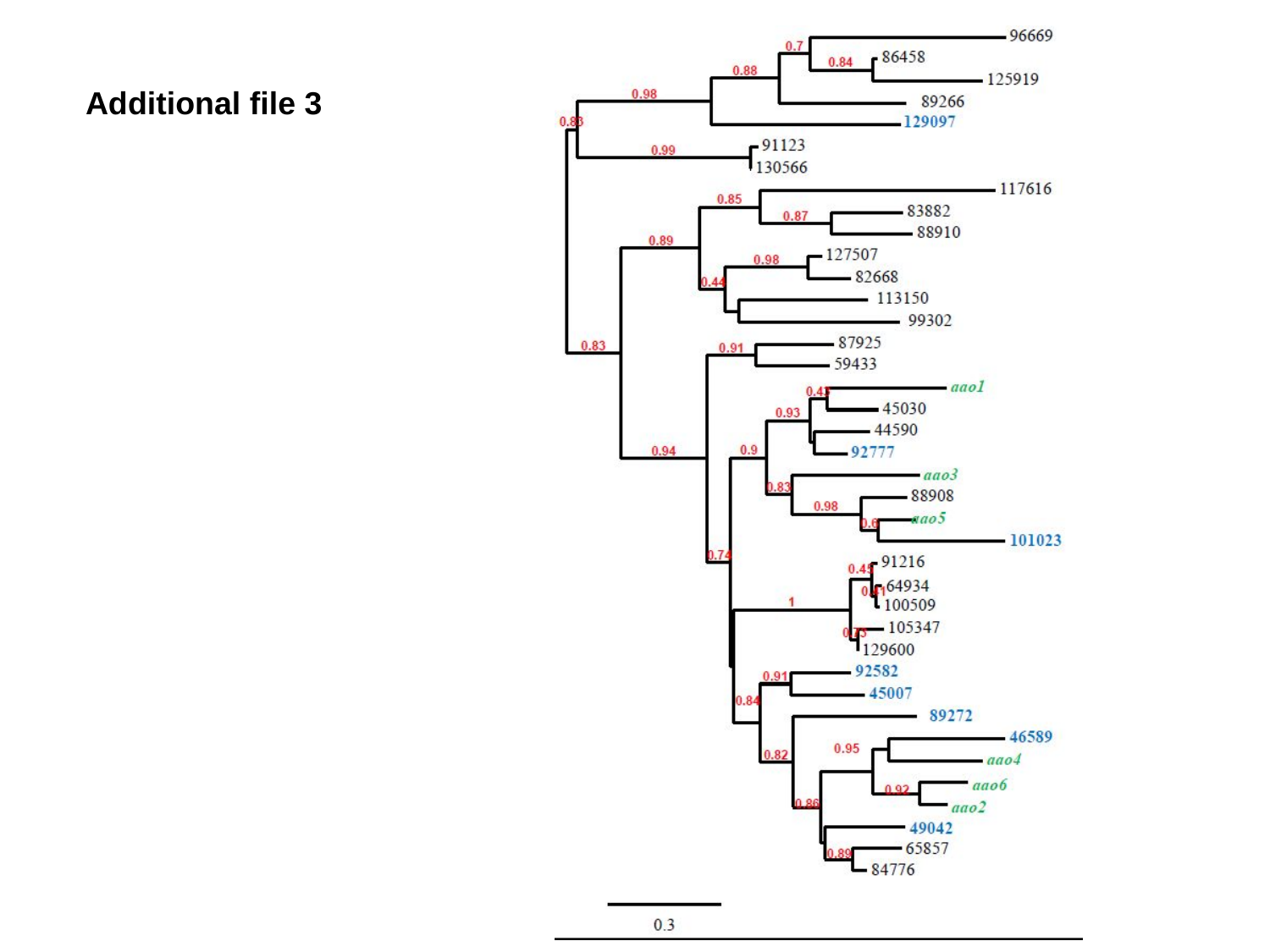

Additional file 3

Supplement: Additional file 3: — Phylogenetic analysis of the AAO family in P. ostreatus. Phylogenetic analysis based on the protein sequences of AAOs from P. ostreatus. Green represents proteins whose abundance increased after HMF addition to the media. Blue represents additional genes whose expression was induced (as determined by real-time PCR; data not shown) 24 h after HMF was added to the medium. [file 13068_2015_244_MOESM3_ESM.pptx]
