# Supplementary material for: Detoxification of 5-hydroxymethylfurfural by the Pleurotus ostreatus lignolytic enzymes aryl alcohol oxidase and dehydrogenase
Source: Biotechnol Biofuels. 2015 Apr 11;8:63. doi: 10.1186/s13068-015-0244-9 (PMC4403834; doi:10.1186/s13068-015-0244-9)
Supplement: Additional file 4: — Primer information. [file 13068_2015_244_MOESM4_ESM.docx]

| **Gene** | **Transcript ID** | **Primer designation** | **Sequence (5′ --->3′)** | **Amplicon (bp)** | **Reference** |
| --- | --- | --- | --- | --- | --- |
| *β-tubulin* | 117235 | tub_117235_F1374 | ACCAGTTCCACCACCAAGAG | 80 | This study |
|  |  | tub_117235_R1506 | TGTTGTGCGTAAGGAAGCTG |  |  |
| *aad1* | 75413 | AAD_75413_F72 | AGGGTATTGTCCCCCAACTG | 130 |  |
|  |  | AAD_75413_R191 | TTTGAAGCTGGCCTCTTTGT |  |  |
| *aao1* | 69649 | AAO_69649_F1495 | GATGCCTCGATCTTCCCATA | 87 |  |
|  |  | AAO_69649_R1688 | CTTAATGTCGTCGGCCATCT |  |  |
| *aao2* | 82653 | AAO_82653_F108 | GTTGTCGGAGAACCTGGGTA | 146 |  |
|  |  | AAO_82653_R234 | AGGCAGCCTGAGGTACTGAA |  |  |
| *aao3* | 93955 | AAO_93955_F1013 | ATCCCCTCCTAGCCTGTGTT | 117 |  |
|  |  | AAO_93955_R1110 | GTCCGGTATGGTTTGCATTC |  |  |
| *aao4* | 114510 | AAO_114510_F375 | GGGATCCTCGGACAACTACA | 115 |  |
|  |  | AAO_114510_R489 | CGAAATGGTGTCGATTTGTG |  |  |
| *aao5* | 116309 | AAO_116309_F1040 | TCTTCGTCAACAGCAACCAG | 129 |  |
|  |  | AAO_116309_R1149 | AGGCCAAGTGGTTAGCAATG |  |  |
| *aao6* | 121882 | AAO_121882_F941 | CTGGCATCGGTGATCCTACT | 101 |  |
|  |  | AAO_121882_R1041 | CACCGCAATGATATGGTCAG |  |  |
|  |  | MnP4R1609 | CAAGTGGGCCGCTCCGAC |  |  |
